# A Framework for Integrating Large Language Models into Memetic Algorithms

**DOI:** 10.3390/biomimetics11060383

**Published:** 2026-06-01

**Authors:** Maxim Sakharov

**Affiliations:** Independent Researcher, Moscow 107143, Russia; maxim@basissoft.ru

**Keywords:** memetic algorithms, global optimization, large language models, code generation, algorithm generation, population-based methods

## Abstract

Memetic algorithms achieve strong optimization performance by combining population-based global search with local refinement operators, yet their effectiveness critically depends on the design and management of memes. Local search strategies are typically handcrafted, problem-specific, and fixed prior to execution. This paper proposes a fourth-generation memetic framework in which Large Language Models (LLMs) are embedded directly into the optimization loop as adaptive generators of local search operators. At each triggering point, the LLM receives a structured state vector encoding the current search dynamics and generates a candidate meme in the form of an executable Python function. Generated operators are subject to a two-stage validation procedure combining semantic similarity assessment and implementation-level comparison, ensuring that only sufficiently novel and syntactically correct operators are admitted to a dynamically growing meme library. A cooldown-regulated triggering mechanism balances periodic and stagnation-based generation, while a probability-weighted selection policy prioritizes newly generated memes without discarding previously validated ones. The proposed framework is evaluated on the CEC 2017 benchmark suite for continuous black-box optimization and compared against classical memetic algorithms. Experimental results demonstrate that the LLM-driven approach consistently outperforms non-LLM memetic baselines, confirming the viability of generative language models as adaptive heuristic components within population-based optimization.

## 1. Introduction

In recent decades, evolutionary population-based algorithms have become powerful and widely adopted tools for solving complex optimization problems. One of the key advantages of this class of methods, in addition to their conceptual simplicity and inherent population diversity, is their high probability of locating so-called suboptimal solutions, that is, solutions situated in the vicinity of the global optimum. In many real-world optimization scenarios, such solutions are sufficient from a practical standpoint [1,2,3].

Despite these strengths, population-based algorithms also exhibit well-known limitations. In particular, their convergence rate often decreases significantly near high-quality solutions, as they generally lack mechanisms for exploiting fine-grained local information about the underlying fitness landscape [4]. This limitation becomes especially important in large-scale and computationally demanding real-world problems, where evaluation budgets are restricted and high solution quality must be achieved within strict time constraints, for example in optical design problems [5,6]. Simply increasing computational power is not sufficient to cope with the growing complexity of modern optimization tasks; instead, qualitatively new algorithmic paradigms are required [7].

Additionally, it has been shown that relying on a single optimization method is often insufficient to obtain high-quality solutions across diverse problem classes [8]. The need for hybrid strategies is well-recognized beyond purely mathematical benchmarks: recent work has demonstrated that combining global metaheuristic search with domain-specific local refinement consistently yields superior results across a range of engineering design and applied optimization problems [9,10,11].

Those observations have motivated the development of hybrid optimization techniques that combine complementary search strategies. Among these, memetic algorithms (MAs) represent one of the most promising and conceptually rich approaches [12]. Memetic algorithms are population-based metaheuristics inspired by neo-Darwinian evolution and the concept of a meme introduced by Dawkins [13], where a meme denotes a unit of cultural information transmitted through imitation and learning. In the context of optimization, a meme corresponds to a local improvement procedure that refines candidate solutions during the evolutionary process. By combining global exploration with local exploitation, memetic algorithms can achieve a synergistic effect that often surpasses the performance of their individual components.

Memetic algorithms have been successfully applied to a wide range of optimization problems in science and engineering [14,15,16]. However, their effectiveness strongly depends on the choice and management of memes, including the selection of local search operators, their frequency of application, and the conditions under which they are activated. Previous studies have demonstrated that meme selection has a decisive impact on the overall performance of memetic algorithms [17].

In this paper, we propose a formal framework aimed at addressing these design challenges by integrating large language models (LLMs) into the memetic search process. The underlying idea was initially introduced in [18] and is supported by numerical results. The present work extends that study by developing a systematic and formal framework that rigorously defines the role of LLMs within memetic algorithms.

The present study addresses the following research questions: (i) whether large language models can be systematically embedded into memetic algorithms as generators of local search operators; (ii) whether such integration leads to measurable improvements in optimization performance; (iii) which components of the proposed framework are critical to its effectiveness; (iv) how generated memes evolve under online selection pressure; and (v) whether the framework generalizes across different population-based optimization algorithms.

The remainder of the paper is organized as follows. Section 2 provides the theoretical background on evolutionary, swarm-based, and memetic algorithms, tracing their development across generations. Section 3 reviews recent advances in the integration of large language models into optimization and algorithm design. Section 4 formulates the optimization problem and introduces the necessary notations and concepts. Section 5 presents the proposed fourth-generation memetic framework in detail, including state vector design, meme generation and novelty filtering, library management, and the complete algorithmic procedure. Section 6 reports the results of computational experiments on the CEC 2017 benchmark suite. Section 7 discusses the findings, examines the sensitivity of the framework to key design choices, and identifies open questions and limitations. Section 8 summarizes the main contributions and outlines directions for future research.

## 2. Memetic Algorithms

Evolutionary population-based algorithms form a broad class of metaheuristics inspired by natural evolution and collective behavior. Representative examples include genetic algorithms, differential evolution, particle swarm optimization (PSO) [19], whale optimization algorithm (WOA) [20] and moth–flame optimization (MFO) [21]. Despite differences in their update mechanisms and metaphors, these methods share a common structure: a population of candidate solutions, iterative fitness evaluation, and stochastic variation operators. Population-based methods are particularly valued for their robustness, flexibility, and ability to explore complex, multimodal search spaces. However, their reliance on indirect information exchange and population-level dynamics often limits their capacity to exploit fine-grained local structure of the objective function. As a consequence, convergence tends to slow significantly in the vicinity of high-quality solutions, especially in high-dimensional or ill-conditioned landscapes.

Memetic algorithms (MAs) extend evolutionary and swarm-based optimization by incorporating local search procedures into the population-based search process [12]. The notion of a meme, originating from Dawkins’ theory of cultural evolution [13], denotes a unit of information transmitted and refined through imitation and learning. In optimization, a meme corresponds to a local improvement operator that refines candidate solutions during the search. By combining global exploration with local exploitation, memetic algorithms often achieve superior convergence behavior and solution quality compared to their non-hybrid counterparts, and their effectiveness has been demonstrated across a wide range of optimization problems. Recent applications span domains including scheduling and manufacturing, structural and mechanical engineering design [22] and energy systems [10].

Nevertheless, their performance critically depends on the design and management of memes including operator selection, application frequency, and activation criteria. A structured taxonomy of memetic algorithms was presented in [23], distinguishing four generations based on increasing levels of algorithmic autonomy. The first generation belongs to the class of hybrid algorithms that couple a population-based global search with a local refinement procedure. These algorithms cannot be unambiguously classified as evolutionary in the Darwinian sense, as they lack mechanisms for transmitting memetic information across generations, selection and mutation of memes, and related principles. Key design questions were how often local search should be applied; to which individuals; for how long; at what computational cost; and which memes are appropriate for a given problem.

The second generation addresses several of these issues through multi-memetic designs that incorporate operations for transmitting and selecting memes. In some variants, memetic information is inherited from parents by offspring; others employ hyper-heuristics or Lamarckian meta-learning [24] to select memes from a competing pool based on past performance.

The third generation introduces self-generation and self-adaptation as local search engines co-evolve with candidate solutions, tracking structural patterns in the search space. Across all three generations, memes function as mechanisms for controlling the evolution of individuals, with their degree of engagement governed by performance indicators and parameters that balance global and local search.

The fourth generation of memetic algorithms is characterized by higher-level cognitive capabilities, including recognition, generalization, and long-term memory, in which memes are not confined to a single problem instance but evolve across runs and adapt to broader contextual information. The concept of a fourth-generation memetic algorithm realized through large language models was introduced by the authors in [18]. In that preliminary study, the primary focus was placed on exploring the feasibility of LLM-driven generation of local search strategies and demonstrating the practical potential of the approach through several proof-of-concept experiments. However, many important methodological and architectural aspects remained open, including the formal organization of meme generation, integration with the optimization process, and systematic evaluation methodology. The present work extends that contribution by providing a complete formal framework, a rigorous algorithmic specification, and an extensive computational validation on standard continuous optimization benchmarks. Beyond empirical evaluation, the paper identifies key design dimensions, open theoretical questions, and concrete directions for future development of this paradigm.

## 3. Related Works

Recent advances in large language models have opened new directions for mathematical optimization, with a growing body of work exploring how LLMs can be embedded into optimization workflows. Rather than a single unified paradigm, this literature reflects several distinct but complementary integration strategies, each illuminating a different facet of the LLM–optimization relationship.

The most foundational question in this area concerns what role an LLM should play within an optimization process. The survey by the authors of [25] provides a systematic answer, positioning LLMs not merely as coding assistants but as integral components that can act simultaneously as optimizers generating and refining candidate solutions, as surrogate predictors estimating solution quality, and as designers proposing entire algorithms or heuristics. The authors argue that the most successful approaches adopt a hybrid paradigm in which LLMs provide semantic reasoning and creative exploration while traditional optimization methods ensure structured search and convergence guarantees—a perspective directly aligned with the use of LLMs for meme generation proposed in the present work. This framing is reinforced by [26], which specifically addresses the limitations of standalone LLM reasoning, such as hallucinations and partial solutions, by grounding generation within an evolutionary framework that provides diversity and convergence control. Taken together, these works establish that neither LLMs nor classical optimizers alone are sufficient: the productive design space lies in their structured combination.

A second cluster of studies moves beyond general principles to demonstrate concrete integrations. Studies [27,28] are particularly relevant to the present work because they embed LLMs directly into memetic algorithm architectures. The framework in [27] integrates LLM-driven heuristic exploration, domain-specific local refinement, and memory-aware reflection, demonstrating that LLMs can serve as interpretable heuristic designers that dynamically evolve strategies by synthesizing natural language and program representations. The work in [28] takes a similar approach for constrained scheduling, embedding LLM-generated heuristic rules and memetic operators within a classical evolutionary local search loop and demonstrating competitive benchmark performance. Both studies confirm that LLMs can reduce the manual effort traditionally required to construct effective memetic operators, but both generate operators in a partially offline or externally guided manner rather than under continuous optimization pressure.

More general evolutionary integrations are explored in [29], which empirically investigates LLMs participating directly in the evolutionary workflow by proposing candidate solutions and variation operators, and in [30], which introduces a bidirectional coupling where evolutionary experience is fed back into the LLM when the search stagnates to produce seed solutions that escape local optima. The latter is notable for its explicit feedback mechanism as the LLM is not merely queried but informed by the state of the search, enabling adaptive behavior across iterations. This bidirectional structure is closest in spirit to the architecture proposed here, though the present work extends it to the generation and online selection of local search operators rather than solution candidates.

A third strand of research elevates the LLM from an operator within an algorithm to a generator of complete algorithms. In [31], the LLM is embedded in an evolutionary loop that generates, mutates, evaluates, and selects entire metaheuristics based on performance criteria, with evolutionary feedback iteratively improving these designs and reducing dependence on human expertise. The study [32] demonstrates a related capability, using GPT-4 Turbo to automatically generate and evolve variants of the Self-Organizing Migrating Algorithm without expert intervention; one LLM-derived variant substantially outperformed the baseline while deviating meaningfully from traditional designs, illustrating that LLM-generated constructs can diverge productively from human-engineered heuristics.

These results are significant because they establish that LLMs are capable of genuine algorithmic creativity rather than mere recombination of known patterns. At the same time, the offline or population-level nature of algorithm generation in these works means the LLM receives limited real-time feedback from individual optimization runs—a constraint the present framework explicitly removes.

Underlying these empirical contributions are a set of theoretical and survey works that provide conceptual grounding. The work [33] draws explicit parallels between LLMs and evolutionary algorithms, comparing token representation to individual encoding and transformer training to parameter adaptation, and argues that shared generative-iterative structures provide a principled basis for hybrid methods. Surveys [25,34] synthesize the broader landscape, analyzing prompt design, automated strategy evolution, and representation choices as central technical challenges. These contributions contextualize the empirical results and support the view that LLM–EA hybrids constitute a coherent and theoretically motivated research direction rather than an ad hoc engineering choice.

Across this body of literature, a common pattern emerges: LLMs are either used offline to design operators or algorithms before optimization begins, or they are embedded as solution proposers that operate at the population level. What has not been demonstrated is a closed-loop architecture in which the LLM generates local search operators during a run, those operators are evaluated under real fitness pressure, and the resulting performance signal is used to guide subsequent LLM proposals. This is precisely the gap the present work addresses.

Unlike approaches in which the LLM generates algorithms offline or serves as a standalone solution proposer, the framework developed here places the LLM inside a continuous feedback loop with the fitness function: it receives structured representations of the current search state, proposes meme operators, and adapts subsequent proposals based on empirical performance. This architecture positions the LLM simultaneously as a creative generator and an adaptive optimization component, enabling the accumulation of transferable meme knowledge across runs which is a direct practical realization of the fourth-generation memetic algorithm paradigm that has previously remained conceptual.

## 4. Problem Formulation

Initially, memetic algorithms were proposed as one of the ways to improve the efficiency of genetic algorithms. However, the combination scheme of global and local search used in MA can be used to construct search algorithms also based on other population algorithms. The term “memetic algorithms” was first proposed by P. Moscato [12] to denote a class of stochastic global search methods that combine the strengths of local search methods, tailored for specific practical problems, and population global search methods.

In this study we use memetic algorithms to solve a global constrained nonlinear minimization problem

(1) $$\underset{X\in D\subset R^{n}}{\min}F(X)=F\left(X^{*}\right)=F^{*}.$$

Here F(X) is the scalar objective function, $X=(x_{1},x_{2},\ldots ,x_{n})$ is the n-dimensional vector of variables, $F\left(X^{*}\right)=F^{*}$ is the required minimum value, $R^{n}$ is the n-dimensional arithmetical space, and D is the constrained search domain

(2) $$D=\{X|x_{i}^{min}\leq x_{i}\leq x_{i}^{max},i\in [1:n]\}\subset R^{n}.$$

In this work F(X) is treated as a black-box function, and the feasible set D is defined by simple bound constraints.

As discussed in [23], memetic algorithms can be organized into four generations of increasing algorithmic autonomy, the first three of which were outlined in the preceding section. Across all three generations, memes serve as mechanisms for controlling individual evolution, with their engagement governed by performance indicators and parameters that balance global and local search.

The fourth generation departs fundamentally from this paradigm. Rather than drawing memes from a predefined pool, memes emerge organically through information exchange among groups of candidate solutions. They can be retained in memory as patterns accumulated from solving prior problems and retrieved through associations between stored patterns and new tasks. This constitutes a qualitatively higher order of learning than local search alone, and it is this generation that the present work formally develops and computationally investigates.

## 5. The Proposed General Framework for LLM-Enhanced Memetic Algorithms

This section presents the formal framework for LLM-enhanced memetic algorithms, in which the on-demand synthesis of memes via external intelligent systems is realized through large language models. Importantly, the LLM does not replace the optimization algorithm but augments it by providing candidate memes conditioned on the current search state. The global search component can be implemented by an arbitrary population-based algorithm, which retains full control over the optimization trajectory, while the LLM serves as a source of adaptive heuristic variations. From this perspective, the framework implements a co-evolutionary mechanism between solutions and memes: the population evolves in the solution space, and the meme library evolves in the space of local search strategies. This distinguishes the fourth generation from earlier approaches, where meme evolution is either absent or restricted to selection among predefined operators. At the same time, the framework preserves a key empirical principle that all generated memes must be validated under real optimization pressure, as there is no guarantee that the LLM produces fundamentally new or effective strategies.

### 5.1. State Representation and Prompt Construction

Within the proposed framework, the state representation plays a fundamentally more important role than in earlier generations of memetic algorithms. In classical and multi-memetic approaches, the choice of local search operator is either deterministic or based on simple heuristics. In contrast, the state vector here becomes the primary source of information driving meme generation: the ability of the LLM to produce meaningful local search operators is directly constrained by the informativeness of this representation.

In the present framework, the state vector captures the current behavior of the optimization process in an online setting, encoding convergence dynamics, population diversity, and search regime characteristics. This formulation allows the LLM to implicitly condition generated memes on different search phases, for example, distinguishing stagnation from high-exploration regimes. It also introduces a potential limitation: if important characteristics of the problem or search dynamics are absent from the state vector, the generated memes may resemble generic local search heuristics, such as hill climbing or adaptive step-size methods [35], rather than strategies tailored to the current landscape. It’s also important to mention that the state vector doesn’t contain any information on the problem under optimization and focuses only on the optimization process and its main features.

The components of $s_{t}$ were selected to provide the LLM with a compact but sufficiently informative characterization of the search process across three complementary axes: convergence dynamics ($I_{t},\kappa_{t}$), population geometry ($D_{t},S_{t},E_{t}$), and fitness landscape structure ($V_{t},L_{t},M_{t}$). Each component was chosen on the basis of computational tractability in an online setting, requiring no additional function evaluations. Alternative features such as gradient approximations were excluded as the framework targets black-box problems where gradients are unavailable. The grouping of components into these three axes also reflects a deliberate design choice: each axis captures a distinct aspect of search behavior that may call for qualitatively different meme strategies.

Let $P_{t}=\{X_{1}(t),\ldots ,X_{N}(t)\}$ denote the population of N candidate solutions at iteration t and let $F_{t}^{*}$ denote the best fitness value observed up to iteration t. The state vector $s_{t}\in R^{8}$ is defined as:

(3) $$s_{t}=(I_{t},\;\kappa_{t},\;D_{t},\;S_{t},\;E_{t},\;V_{t},\;L_{t},\;M_{t})$$

The improvement rate $I_{t}$ measures the relative change in best fitness between consecutive iterations:

(4) $$I_{t}=\frac{F_{t-1}^{*}-F_{t}^{*}}{\left|F_{t-1}^{*}\right|+\varepsilon }$$

where ε>0 is a small constant ensuring numerical stability. Positive values indicate improvement; zero indicates stagnation. The stagnation counter $\kappa_{t}$ records the number of consecutive iterations without improvement:

(5) $$\kappa_{t}=\left\{\begin{array}{c} 0,\;if\;F_{t}^{*}<F_{t-1}^{*}\\ \kappa_{t-1}+1,\;otherwise \end{array}\right.$$

Together $I_{t}$ and $\kappa_{t}$ provide complementary information about convergence dynamics. $I_{t}$ captures the instantaneous rate of progress, while $\kappa_{t}$ accumulates evidence of prolonged stagnation that a single-iteration metric would fail to detect.

The population diversity $D_{t}$ quantifies the spatial spread of the population as the mean Euclidean distance from each individual to the population centroid

(6) $$D_{t}=\frac{1}{N}\sum_{i=1}^{N}{\left\|X_{i}\left(t\right)-\bar{X(t)}\right\|}_{2}$$

The mean step size $S_{t}$ measures the average displacement of individuals between consecutive iterations:

(7) $$S_{t}=\frac{1}{N}\sum_{i=1}^{N}{\left\|X_{i}\left(t\right)-\bar{X_{i}(t-1)}\right\|}_{2}$$

The exploration-exploitation ratio $E_{t}$ characterizes the balance between spatial spread and movement dynamics:

(8) $$E_{t}=\frac{D_{t}}{D_{t}+S_{t}+\varepsilon }$$

Values of $E_{t}$ close to 1 indicate that the population is spread widely relative to its movement, suggesting an exploration-dominated regime; values close to 0 indicate convergence or exploitation-dominated dynamics. Jointly, $D_{t},\;S_{t}$ and $E_{t}$ provide a multi-scale description of population geometry: $D_{t}$ captures the global extent of the search cloud, $S_{t}$ reflects the velocity of individual trajectories, and $E_{t}$ summarizes their relative balance.

The fitness variance $V_{t}$ measures the dispersion of objective values across the population:

(9) $$V_{t}=\frac{1}{N}\sum_{i=1}^{N}{\left(F\left(X_{i}\left(t\right)\right)-\bar{F_{t}}\right)}^{2},\;\bar{F_{t}}=\frac{1}{N}\sum_{i=1}^{N}F\left(X_{i}\left(t\right)\right)$$

The local fitness dispersion $L_{t}$ captures fine-grained fitness heterogeneity by computing the mean absolute fitness difference between each individual and its nearest neighbor:

(10) $$L_{t}=\frac{1}{N}\sum_{i=1}^{N}\left|F\left(X_{i}\left(t\right)\right)-F\left(X_{nn(i)}\left(t\right)\right)\right|$$

where $nn\left(i\right)=\arg\underset{i\neq j}{\min}{\left\|X_{i}\left(t\right)-X_{j}\left(t\right)\right\|}_{2}$ denotes the nearest neighbor of individual i in the population. The pair $V_{t}$ and $L_{t}$ provides complementary views of fitness heterogeneity: $V_{t}$ reflects global spread across the entire population, whereas $L_{t}$ is sensitive to local fitness ruggedness in the immediate neighborhood of each individual.

Finally, the multimodality proxy $M_{t}$ estimates the degree of multimodality in the current population as the fraction of individuals that are locally non-minimal, in other words, worse than at least one of their k nearest neighbors:

(11) $$M_{t}=\frac{1}{N}\sum_{i=1}^{N}1\left[\exists \;j\in N_{k}\left(i\right):F\left(X_{j}\left(t\right)\right)<F\left(X_{i}\left(t\right)\right)\right]$$

where $N_{k}\left(i\right)$ denotes the set of k nearest neighbors of individual i, and k is a small, fixed integer (here k=3). High values of $M_{t}$ suggest that the population spans multiple distinct basins of attraction, which in turn informs the LLM that escape-from-basin strategies may be more appropriate than fine-grained local refinement.

The state vector $s_{t}$ serves as the basis for constructing the meme generation prompt. This step is critical: the prompt content directly conditions the LLM’s output, and its quality determines whether the generated operator reflects the current search context or defaults to generic heuristic patterns. The meme generation mechanism is embedded directly into the optimization loop through a fixed prompt template. The LLM is tasked with generating a local search operator in the form of a deterministic Python function that performs a fixed number of improvement steps on a single candidate solution.

A key distinction of the fourth-generation framework is that meme generation is not a one-time offline process but an iterative, state-dependent procedure: at each triggering point, the LLM produces a candidate operator conditioned on the current $s_{t}$. This significantly increases the diversity of potential memes, while also raising the question of whether the generated operators represent genuinely novel strategies or variations in known methods. This question is also addressed through empirical validation within the optimization loop. The prompt template used in the present framework takes the following form:

*We are running a population-based continuous optimization algorithm*.

*A local search operator (meme) is required.*

*Current optimization state:*

*Improvement rate   (I_t): {I_t}*

*Stagnation counter  (κ_t): {κ_t}*

*Population diversity  (D_t): {D_t}*

*Mean step size    (S_t): {S_t}*

*Exploration-exploitation ratio (E_t): {E_t}*

*Fitness variance    (V_t): {V_t}*

*Local fitness dispersion (L_t): {L_t}*

*Multimodality proxy  (M_t): {M_t}*

*Goal:*

*Generate a Python function that performs 30 local improvement steps*

*on a single solution vector x.*

*Requirements:*

*1. Function signature must be:*

*def generated_meme(x, objective_function, lower_bound, upper_bound):*

*…*

*2. Input:*

*- x: numpy array of shape (d,)*

*- objective_function: callable f(x)*

*- lower_bound: numpy array (d,)*

*- upper_bound: numpy array (d,)*

*3. The function must:*

*- Modify x locally (small perturbation or structured step)*

*- Respect bounds*

*- Return (x_new, f_new)*

*4. Do NOT use:*

*- external libraries except numpy*

*- randomness without numpy*

*- recursion*

*- printing*

*- global variables*

*Return only valid Python code.*

*Do not include explanations.*

### 5.2. Novelty Assessment and Meme Library Management

The generated meme is evaluated in terms of structural and semantic novelty prior to integration into the optimization loop. Since there is no guarantee that the LLM does not reproduce previously generated algorithms or their close variations, the framework explicitly incorporates a similarity filtering stage. The concept of structural similarity control was introduced in [36].

Novelty is assessed through a hybrid similarity measure combining two complementary methods. Semantic similarity is computed using GraphCodeBERT [37], which produces code embeddings that capture high-level algorithmic intent. Implementation-level similarity is measured using the Jaccard coefficient [38] over tokenized operator representations. This dual approach addresses a challenge in optimization algorithm assessment; specifically, while GraphCodeBERT provides robust general-purpose code understanding, its embeddings may not always capture mathematically significant variations characteristic of optimization routines, for example, differences in weight update schemes or step-size adaptation logic. Conversely, Jaccard similarity at the token level is particularly effective at detecting trivial modifications that preserve overall structure while altering surface syntax.

Formally, let $\sigma_{sem}(m,m^{\prime })$ and $\sigma_{impl}(m,m^{\prime })$ denote the GraphCodeBERT cosine similarity and Jaccard similarity between candidate meme m and library meme $m^{\prime }$ respectively. A candidate meme m is considered similar to an existing library meme $m^{\prime }$ if either similarity exceeds a fixed threshold τ=0.9. This threshold was selected empirically [36] as a conservative value that permits structural variation while filtering near-duplicate operators.

(12) $$sim\left(m,m^{\prime }\right)=1\left[\sigma_{sem}\left(m,m^{\prime }\right)>\tau \cup \sigma_{impl}\left(m,m^{\prime }\right)>\tau \right]$$

If $sim\left(m,m^{\prime }\right)=1$ for any $m^{\prime }\in L_{t}$, where $L_{t}$ denotes the current meme library, the candidate is rejected and the selection falls back to the existing library. Otherwise, the candidate is accepted and added to $L_{t}$. It should be noted that structural differences do not necessarily imply conceptual novelty. An LLM-generated operator may be distinct in implementation while still being interpretable as a variant of a known method, for example, hill climbing with adaptive step size. The evaluation mechanism partially mitigates this issue by focusing on empirical performance rather than theoretical novelty: each candidate meme is tested within the optimization loop, and only those demonstrating measurable improvement over the current best solution are retained in $L_{t}$.

At each triggering point (every Δ iterations), the active meme is selected according to the following policy. If a novel meme $m_{new}$ has been accepted, it is assigned selection probability $1-|L_{t}|\cdot \theta$, while each meme $m^{\prime }\in L_{t}$ retains a small probability θ:

(13) $$P\left(m\right)=\left\{\begin{array}{c} 1-\left|L_{t}\right|\cdot \theta ,\;if\;m=m_{new}\\ \theta ,\;if\;m\in L_{t} \end{array}\right.$$

where θ is a fixed exploration probability. If no novel meme was generated at the current trigger, either due to similarity rejection or LLM unavailability, selection is performed uniformly over $L_{t}$. This policy ensures that newly generated operators are prioritized while preserving access to previously validated strategies, maintaining both adaptability and continuity throughout the optimization process.

The proposed framework maintains a dynamically growing library $L_{t}$ that accumulates operators generated and validated during the optimization run. This mechanism can be interpreted as within-run learning: rather than discarding memes after use, the framework retains those that proved effective and makes them available for reuse in subsequent iterations. The library thus gradually captures a problem-specific portfolio of local search strategies that are empirically suited to the current objective landscape.

An important design decision is that the meme library is not intended to persist across optimization runs. Since different problem instances induce different search dynamics, memes effective for one instance are not assumed to transfer to another. Each run begins with an empty library and constructs its own portfolio from scratch, driven by the evolving state representation. This distinguishes the present framework from transfer optimization or algorithm selection approaches, where cross-instance knowledge reuse is an explicit goal.

### 5.3. General Framework

The integration of LLMs introduces inference latency and computational overhead not presented in earlier generations of memetic algorithms. The proposed framework mitigates this through the reuse of previously generated memes: as optimization progresses and $L_{t}$ grows, the frequency of LLM calls decreases and the algorithm increasingly operates using the accumulated library. This behavior represents a form of knowledge consolidation, where the upfront cost of meme generation is amortized over repeated reuse. Practical challenges remain, including the handling of syntactically invalid or semantically degenerate operators and the latency associated with LLM inference. These issues highlight the need for robust validation mechanisms and efficient integration strategies. From the perspective of memetic algorithm evolution, the proposed approach represents a shift from static or manually designed hybridization toward autonomous construction of hybrid strategies, in which the practitioner defines the overall framework while specific local search behaviors emerge through generation, evaluation, and selection. At the same time, the empirical foundation of memetic algorithms is fully preserved: all candidate strategies must demonstrate their effectiveness through direct evaluation before contributing to the optimization process.

Algorithm 1 summarizes the complete execution flow of the proposed framework. The algorithm proceeds in three interleaved phases at each iteration: global search update, meme generation and filtering, and local refinement. The global search component is treated as a black box, allowing any population-based algorithm to serve as the underlying optimizer. Meme generation is triggered periodically or upon stagnation detection, subject to the library capacity constraint $L_{max}$. Generated memes pass through a two-stage validity check (syntactic correctness followed by novelty filtering) before being admitted to the library L. Local refinement is applied to the top 0.15·N individuals ranked by fitness, with each selected individual undergoing up to η=30 improvement steps or until local stagnation is detected. The meme applied to each individual is drawn from the current library according to a selection policy that prioritizes the most recently accepted meme while preserving access to all previously validated operators.
**Algorithm 1.** Fourth-Generation Memetic Algorithm Framework with LLM-driven Meme Generation**Input:** objective function F, bounds D, population size N, max iterations T, trigger interval Δ, stagnation threshold λ, library capacity $L_{max}$, meme steps η, meme stagnation limit ρ, similarity threshold τ, library exploration parameter θ **Output:** best solution $X^{*}$, meme library L $P_{0}\leftarrow \mathrm{InitializePopulation}\;\left(N,D\right)$$F^{*}\leftarrow {min}_{i}F\left(X_{i}^{\left(0\right)}\right)$, $x^{*}\leftarrow \arg{min}_{i}F\left(X_{i}^{\left(0\right)}\right)$L←∅, κ←0, $m_{new}\leftarrow \emptyset$$t_{last}\leftarrow \delta$ **for** t=1,…,T **do**   $P_{t}\leftarrow \mathrm{GlobalSearchUpdate}\left(P_{t-1}\right)$   Update $F^{*}$, $X^{*}$, $\kappa_{t}$   Compute state vector $s_{t}=\left(I_{t},\kappa_{t},D_{t},S_{t},E_{t},V_{t},L_{t},M_{t}\right)$    // Meme Generation Trigger    **if** $\left(t\;mod\;\Delta =0\right.$ **or** $\left.\kappa_{t}>\lambda \right)$ **and** $|L|<L_{max}$ **and** $t-t_{last}\geq \delta$ **then**       $m_{cand}\leftarrow \mathrm{LLM}-\mathrm{GenerateMeme}\left(s_{t}\right)$       $t_{last}\leftarrow t$       **if** $m_{cand}$ is syntactically valid and executes without runtime error **then**         **if** $∄\;m^{\prime }\in L$ s.t. $\sigma_{sem}(m_{cand},m^{\prime })>\tau$ **or** $\sigma_{impl}(m_{cand},m^{\prime })>\tau$            $L\leftarrow L\cup \left\{m_{cand}\right\}$,            $m_{new}\leftarrow m_{cand}$         **else**            $m_{new}\leftarrow \emptyset$// rejected due to similarity         **end if**       **else**         $m_{new}\leftarrow \emptyset$// rejected due to invalid or erroneous code       **end if**   **end if**    // Meme Selection    **if** L≠∅      **if** $m_{new}\neq \emptyset$        Select $m^{*}$ according to:          $P\left(m^{*}=m\right)=\left\{\begin{array}{cc} 1-(|L|-1)\cdot \theta & \mathrm{if}\;m=m_{new}\\ \theta & \mathrm{if}\;m\in L\setminus \{m_{new}\} \end{array}\right.$        where $\theta <\frac{1}{|L|-1}$ to ensure $P\left(m^{*}=m_{new}\right)>0$      **else**        Select $m^{*}$ uniformly from L       **end if**        // Meme Application to Top 15%        $E_{t}\leftarrow \mathrm{top}\;\left\lceil 0.15\cdot N\right\rceil \;\mathrm{individuals}\;\mathrm{of}\;P_{t}\;\mathrm{ranked}\;\mathrm{by}\;f$       **for each** $X\in E_{t}$ **do**         $\kappa_{local}\leftarrow 0$, $X_{cur}\leftarrow x$         **for** s=1,…,η **do**            $\left(X_{new},F_{new})\leftarrow m^{*}(X_{cur},F,D\right)$            **if** $F_{new}<F(X_{cur})$               $X_{cur}\leftarrow X_{new}$, $\kappa_{local}\leftarrow 0$            **else**               $\kappa_{local}\leftarrow \kappa_{local}+1$            **end if**            **if** $\kappa_{local}\geq \rho$
**break end if**         **end for**         **if** $F(X_{cur})<F(X)$ **then**             Replace X in $P_{t}$ with $X_{cur}$             Update $F^{*}$, $X^{*}$ if improved         **end if**      **end for**   **end if**   $m_{new}\leftarrow \emptyset$// reset for next iteration**end for****return** $X^{*}$, L

Generated operators that fail syntactic validation or produce a runtime error upon execution are discarded immediately and do not enter the library. In practice, code generation errors are rare with current state-of-the-art large language models; however, error rates vary across models of different capability and architecture, and a systematic characterization of code validity as a function of model choice represents a separate line of investigation beyond the scope of the present work. To prevent redundant LLM calls when both the periodic and stagnation-based triggers coincide, a cooldown parameter δ is introduced. Following any LLM call at iteration $t_{last}$, the next call is suppressed until $t-t_{last}\geq \delta$, regardless of which trigger condition is satisfied. Setting δ=Δ recovers the most conservative behavior, in which the stagnation trigger can only accelerate a scheduled call but cannot introduce additional ones. Smaller values of δ<Δ allow faster response to stagnation while still preventing back-to-back calls. Prior to the first successful meme admission, the library remains empty and the algorithm operates as a standard global search method without local refinement.

**Figure 1:**
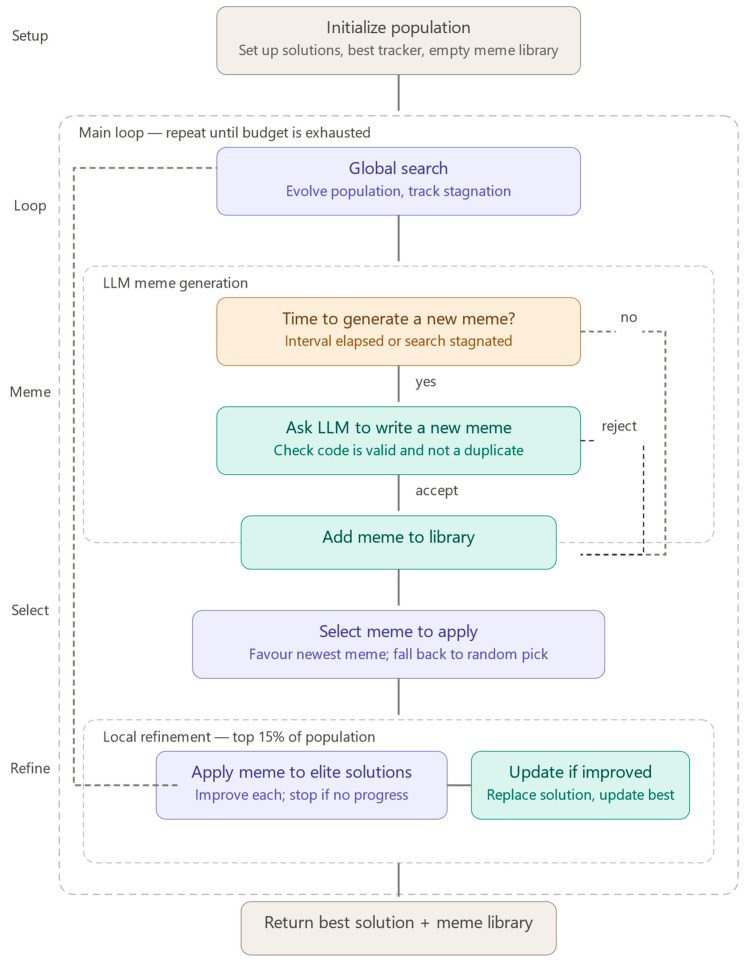
The block diagram of the proposed framework.

The detailed description of the proposed framework is presented below and in Figure 1.

## 6. Experimental Study

### 6.1. Experimental Setup

In order to evaluate the performance of the proposed framework we use a set of high-dimensional benchmark functions from CEC 2017, which was designed single objective real-parameter numerical optimization problems [39]. The present study focuses on the 30-dimensional benchmark setting (n=30) in order to establish and validate the proposed framework. Investigation of scalability properties for higher-dimensional optimization problems will constitute an important next stage of research following the formalization of the proposed paradigm. All algorithms were implemented using Python programming language, while computational experiments were launched on MacBook Pro with 2.3 GHz Quad-Core Intel Core i7 and 32 GB RAM.

Particle Swarm Optimization and the Whale Optimization Algorithm were selected as the underlying global search components for several reasons. Both represent well-established population-based metaheuristics with qualitatively distinct search mechanisms: PSO relies on velocity-based social learning, while WOA employs encircling and spiral-based prey-chasing behaviors. This diversity ensures that conclusions regarding the proposed memetic framework are not tied to a single search paradigm but reflect its behavior across qualitatively different global optimizer dynamics.

Both algorithms have been extensively benchmarked on CEC test suites, providing reliable baseline references against which the contribution of the memetic component can be cleanly isolated. Eight algorithmic variants were evaluated: PSO, WOA, PSO-NM, WOA-NM, MMPSO, MMWOA, AIM-PSO and AIM-WOA. The baseline algorithms (PSO and WOA) use standard metaheuristic search without local refinement. The single-meme variants (PSO + NM and WOA + NM) augment the global search with Nelder–Mead local search [40], representing a first-generation memetic configuration. The multi-memetic variants (MMPSO and MMWOA) randomly select between two local search operators: Nelder–Mead and Random Walk Local Search [41]. The AI-memetic variants (AIM-PSO and AIM-WOA) constitute the proposed fourth-generation framework, employing LLM-generated operators selected according to the state-dependent policy described in Section 5, with the meme library growing dynamically over the course of each optimization run.

All algorithms used a maximum of $T_{max}=2000$ iterations with population size N=30. PSO parameters were set to inertia weight ω=0.7298 and cognitive and social coefficients $c_{1}=c_{2}=1.49618$ following the canonical parameterization of [19]. For WOA, default parameter settings from the original formulation [20] were retained. In all memetic variants, local search operators were applied every 50 iterations to the top 15% of individuals ranked by current fitness, with each application performing up to $\lambda_{max}=30$ local iterations subject to the early termination criterion described in Section 5. All experiments employed 30 independent runs per function.

For the meme generation the GPT-5.3 model was used. The model temperature was set to 0.7 to balance operator diversity with coherence; full determinism via seed was not enforced as stochastic variation across meme proposals is a deliberate feature of the framework rather than a confound. All other parameters were left at API defaults. Each run issued at maximum 40 LLM calls (on average 37 calls when the stagnation criteria is not met) with individual inference latency averaging 2.4 s, contributing approximately 100 s of LLM overhead per run. This represents roughly 14% of total wall-clock time, indicating that inference cost does not constitute a prohibitive bottleneck.

All results obtained are presented in Table 1 and in Figure 1.

### 6.2. Statistical Analysis of the Results

The performance of all optimization strategies was evaluated across 29 benchmark functions, with statistical significance assessed using the Friedman test and pairwise Wilcoxon post hoc comparisons. The Friedman test confirmed highly significant differences among the algorithms ($\chi^{2}=121.98,\;p=2.96\times 10^{-23}$), justifying a detailed pairwise analysis.

The mean Friedman ranks revealed a clear performance hierarchy, with AIM-PSO achieving the best average rank of 1.86, closely followed by AIM-WOA at 2.34. Both LLM-based algorithms substantially outperformed all other competitors, reflecting the advantage of the proposed framework. Notably, the pairwise Wilcoxon test found no statistically significant difference between these two methods (p=0.686), suggesting that, at this level of algorithmic sophistication, the choice of the underlying global search engine, PSO or WOA, has a negligible impact on overall performance. The slightly higher win count of AIM-WOA over AIM-PSO is tentatively attributed to WOA’s more diverse exploration pattern, which provides the adaptive meme selection mechanism with a broader variety of candidate solutions to refine; a thorough investigation of this interaction is left for future work.

Multi-memetic PSO ranked third (3.24) performing significantly worse than both LLM-based variants (p<0.05) but significantly better than all multistart and Nelder–Mead-based strategies. This indicates that while a fixed multi-meme strategy still benefits from diversity in local search, it cannot fully match the flexibility offered by adaptive meme selection. Multi-memetic WOA, however, ranked considerably lower (5.34), suggesting that the WOA global search mechanism interacts less favorably with a fixed multi-meme framework compared to PSO. The Nelder–Mead hybridizations (PSO-NM and WOA-NM) ranked fourth and sixth respectively, performing poorly despite incorporating a classical local search procedure. This can be attributed to the rigidity of the Nelder–Mead simplex method, which, while effective in smooth unimodal landscapes, struggles to provide consistent improvement across the diverse and multimodal benchmark functions used in this study.

The two simple multi-start strategies (PSO and WOA) ranked last overall, with average ranks of 6.41 and 6.51 respectively. Their high mean and maximum objective values, alongside large standard deviations, confirm that repeated independent restarts without any local refinement are insufficient to compete with hybridized approaches. Interestingly, no statistically significant difference was found between multi-start WOA and WOA-NM (p=0.565), suggesting that a single fixed local search operator offers little practical benefit over pure multi-start in this setting.

In terms of pairwise win counts, AIM-WOA achieved the strongest overall performance with 15 wins (51.7% of benchmark functions), followed by AIM-PSO with 8 wins (27.6%), collectively accounting for the majority of pairwise comparisons. These results confirm that the advantage of the proposed framework is not contingent on the choice of the underlying global search algorithm: both PSO-based and WOA-based instantiations of the fourth-generation memetic framework outperform their respective non-LLM counterparts including pure baselines, single-meme variants, and randomly selecting multi-memetic variants. This demonstrates that LLM-driven adaptive meme generation constitutes a genuine and transferable improvement over simpler memetic strategies.

To assess practical significance, Kendall’s W was computed for the global Friedman test, confirming a large overall effect of algorithm choice across the 29 benchmark functions. Pairwise effect sizes were quantified using rank-biserial correlation r. The largest effects were observed between the AIM algorithms and the multistart baselines (r=0.581−0.665), as well as against the Nelder–Mead hybrids (r=0.567−0.636), all qualifying as large effects. In contrast, the comparison between AIM-PSO and AIM-WOA yielded a negligible effect (r = 0.069), confirming that their statistically equivalent ranking reflects a genuinely marginal practical difference. The multistart strategies were mutually negligible (r ≤ 0.030), further confirming their practical equivalence.

**Figure 2:**
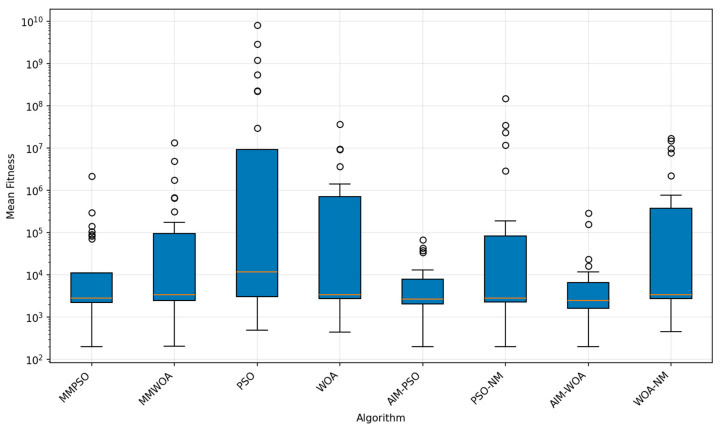
Distribution of objective function values for all algorithms across all benchmark functions and all runs.

Overall algorithm performance comparison is also presented in Figure 2. It shows that the proposed LLM-based memetic approaches produce substantially lower variance and more stable optimization performance compared to the multi-start and classical hybrid methods. In particular, AIM-PSO and AIM_WOA exhibit narrower interquartile ranges and fewer extreme outliers, indicating consistent convergence behavior across all runs and all benchmark functions. In contrast PSO and WOA demonstrate very high variability, with fitness values spanning several orders of magnitude and numerous extreme outliers, reflecting unstable convergence and strong sensitivity to initialization. Overall, the proposed framework significantly improves robustness and reduce performance dispersion.

### 6.3. Meme Similarity Analysis

Analyzing the structure and diversity of LLM-generated memes provides insight into the behavior of the generation mechanism and the degree of novelty achieved by the framework. For this purpose, memes were collected across three representative benchmark functions spanning different landscape classes: $F_{7}$ (multimodal), $F_{15}$ (hybrid) and $F_{25}$ (composite). For each function, the first and last memes admitted to the library were retained for analysis, yielding six memes in total: $m_{1}^{7}$, $m_{3}^{7}$, $m_{1}^{15}$, $m_{3}^{15}$, $m_{1}^{25}$, $m_{3}^{25}$.

**Figure 3:**
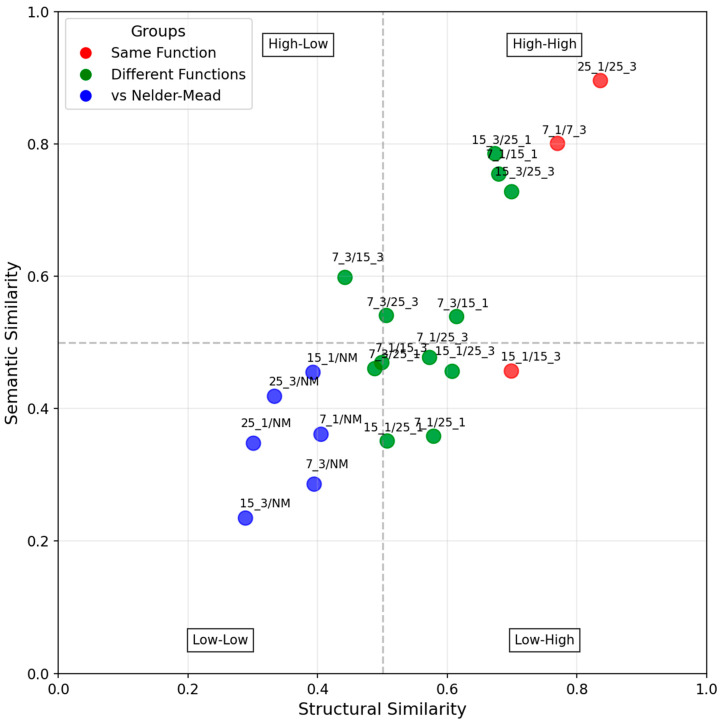
Similarity analysis of the results for the first and the last memes generated for functions F7, F15, F25 and their comparison with the Nelder–Mead method.

Pairwise similarity was assessed according to the method described in Section 5.2. The results are presented in Figure 3. Several observations emerge from the analysis. For $F_{25}$, the first and last generated memes exhibit near-identical similarity scores, despite having been produced in independent runs. This finding highlights the stability of the LLM conditioned on the $s_{t}$: when the search dynamics encoded in the state vector are similar across runs, the model consistently produces structurally comparable operators. For $F_{7}$ and $F_{15}$, by contrast, the first and last memes are noticeably dissimilar, reflecting the evolution of the search state over the course of the optimization and confirming that the generation mechanism responds meaningfully to changes in $s_{t}$. Cross-function comparisons reveal occasional structural overlap between memes generated for different benchmark classes, notably between $m_{3}^{15}$ and $m_{3}^{25}$, and between $m_{1}^{7}$ and $m_{1}^{15}$. This suggests that certain search regimes, as characterized by similar state vectors, produce comparable operator responses from the LLM regardless of the underlying function class.

Nevertheless, the majority of cross-function meme pairs exhibit low similarity scores on both semantic and implementation metrics, indicating that the generation process is broadly sensitive to landscape-specific search dynamics. To establish an external reference point, all six memes were additionally compared against the Nelder–Mead method. The resulting similarity scores are uniformly low across both metrics, confirming that the LLM-generated operators are structurally distinct from this classical local search baseline.

**Figure 4:**
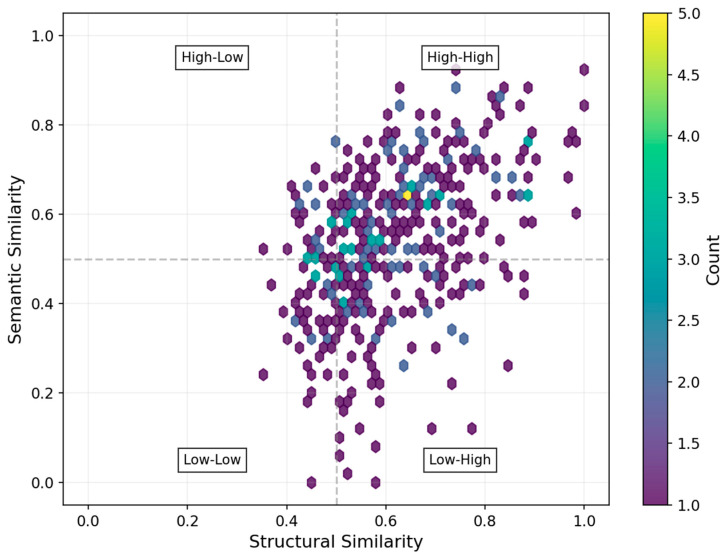
Distribution of pairwise comparisons for 30 randomly selected generated memes.

Additonally, in Figure 4 we present the distribution of the pairwise comparison of generated memes based on 30 randomly selected memes but no more than 2 for every benchmark function. The obtained distirbution supports the central hypothesis of the framework; specifically, large language models, conditioned on a structured representation of the current search state, are capable of generating genuinely novel local search strategies that extend beyond the repertoire of established optimization heuristics.

### 6.4. Meme Efficiency Analysis

In order to further evaluate the quality of the generated memes a set of experiments was carried out with a use of 30 randomly selected previously generated memes. To make the comparison fair, we use a different set of benchmark functions and not the ones some of those memes were generated for. For this study five 30-dimensional traditional benchmarks were used: Sphere, Rastrigin, Ackley, Griewank, and Levy [42] with 20 independent runs from random initial points. Each meme was launched for the exact number of 30 iterations like in the earlier experiments. The memes were ranked by a composite score weighting mean fitness (0.7) and standard deviation (0.3).

The results (Table 2) reveal substantial performance variation across generated operators, with composite scores ranging from 0.044 to 0.841. The top-ranked operators ($m_{1}^{13}$, $m_{1}^{1}$, $m_{3}^{15}$) achieve average values close to the known optima while maintaining low mean errors across multimodal benchmarks. By contrast, the lowest-ranked operators ($m_{3}^{25}$, $m_{3}^{9}$ $m_{3}^{17}$) consistently fail to reach acceptable solutions on any benchmark with high variance. A notable pattern is the systematic advantage of memes generated in the beginning of the optimization (lower index 1) over ones generated during the later stages (lower index 3). This is expected as those memes were generated for a broader exploration while those generated later were for fine tuning the small neighborhood.

The observed variation in meme performance is expected: operators are generated for specific stages of the optimization process and tailored to particular search contexts, not designed for universal benchmark efficiency. This context-specificity is a fundamental feature of the proposed framework, distinguishing it from approaches where operators are selected based on historical performance.

## 7. Discussions

### 7.1. State Vector Design and Its Influence on Meme Quality

The state vector $s_{t}$ constitutes the sole source of contextual information available to the LLM at the point of meme generation, and its design is therefore a critical determinant of the quality and diversity of generated operators. The eight metrics adopted in the present framework capture complementary aspects of search dynamics: convergence rate, stagnation, spatial spread, movement velocity, fitness heterogeneity, and landscape multimodality. They were selected to provide a compact yet informative description of the current optimization regime. Nevertheless, the choice of these particular metrics represents one of many possible state encodings, and the question of optimal state vector design remains open. Several alternative or complementary representations merit investigation. Problem-specific features, such as estimated gradient information, condition number approximations, or basin-of-attraction indicators could provide the LLM with richer landscape context. Dimensionality-aware metrics may also be important: the current formulation does not explicitly encode the problem dimension n, which influences the expected behavior of perturbation-based local search operators. Furthermore, the metrics are computed at the population level and may obscure individual-level variation; sub-population statistics or clustering-based descriptors could capture finer-grained search structure. A systematic ablation study varying the composition of $s_{t}$ would clarify which components most strongly influence the quality of generated memes, and whether a reduced or augmented state representation could improve overall framework performance.

### 7.2. The Role of the LLM and Prompt Sensitivity

The framework treats the LLM as a black-box code generator conditioned on $s_{t}$ and its behavior is therefore subject to all known properties and limitations of large language models. Several dimensions of LLM influence warrant explicit discussion. First, the choice of model is likely to affect both the diversity and the quality of generated operators. More capable models may produce structurally more varied and mathematically sophisticated local search strategies, while smaller or quantized models may default to simpler patterns. The present framework imposes no constraints on the underlying model, but empirical evaluation across models of varying capability would clarify the sensitivity of framework performance to this choice. Second, prompt design introduces a source of variability that is difficult to control systematically. The fixed prompt template adopted here encodes the state vector as a structured list of named scalar values. Alternative representations, like natural language descriptions of the current search regime or few-shot examples of effective operators observed in similar states, may elicit qualitatively different and potentially more effective responses. Prompt engineering for optimization contexts remains an underexplored area with significant practical implications. Third, LLMs are inherently stochastic: repeated calls with identical prompts may produce different operators. This non-determinism can be advantageous from a diversity standpoint but complicates reproducibility. Strategies for controlling or exploiting LLM stochasticity within the optimization loop for example, through temperature scheduling or ensemble generation followed by internal selection, represent a natural extension of the present framework.

### 7.3. Meme Library Dynamics and Selection Policy

The meme library L accumulates operators that have passed novelty filtering, but its growth dynamics and the quality of its contents depend on several interacting design choices. The library capacity $L_{max}$ controls the total number of LLM calls and determines the point at which the algorithm transitions to a purely library-based mode. A small $L_{max}$ limits adaptability; a large $L_{max}$ increases inference cost and may dilute the library with redundant operators if the novelty threshold τ is insufficiently strict. The current selection policy treats all library memes as equally probable alternatives to the newly accepted operator, assigning each a fixed probability θ. This formulation ignores the accumulated performance history of individual memes. A natural extension would weight library memes by their empirical success rate, for example, the fraction of applications that yielded fitness improvement, introducing a form of online reinforcement that gradually concentrates selection probability on the most effective operators.

### 7.4. Generalization, Transferability, and the Scope of the Meme Library

A deliberate design decision in the present framework is that the meme library does not persist across optimization runs. This choice is motivated by the observation that different problem instances induce qualitatively different search dynamics reflected in their respective state vectors and that operators effective for one landscape may be ineffective or counterproductive for another. Each run therefore constructs its own problem-specific portfolio of local search strategies from scratch. At the same time, this decision forecloses the possibility of cross-instance knowledge transfer, which represents a potentially valuable capability aligned with the fourth-generation memetic paradigm. If state vectors from previous runs were used as retrieval keys in a persistent memory structure, previously validated memes could be reused when a new run enters a similar search regime. Whether such transfer would be beneficial in practice depends on the degree to which $s_{t}$ captures transferable landscape features rather than instance-specific dynamics. This question connects the present framework to the broader literature on algorithm selection, meta-learning and transfer optimization, and represents a promising direction for future work.

### 7.5. Limitations and Open Questions

Several limitations of the present framework should be acknowledged explicitly. The similarity filtering mechanism relies on fixed thresholds τ=0.9 for both GraphCodeBERT cosine similarity and Jaccard coefficient. These values were selected empirically and may not generalize across problem classes or LLMs: a threshold appropriate for one model’s output distribution may be too permissive or too strict for another. Adaptive or data-driven thresholds represent a natural improvement direction. However, it should be noted that in the experiments conducted, no acute sensitivity to this parameter was observed across the tested range, providing pragmatic support for the chosen value.

The framework applies the selected meme to the top 15% of the population by fitness, focusing local refinement on the most promising region of the search cloud. While this is consistent with standard memetic practice, it may reduce population diversity over time, particularly in multimodal landscapes where lower-fitness individuals occupy distinct basins of attraction. Alternative application strategies such as fitness-proportional selection, diversity-aware targeting, or periodic application to randomly selected individuals could mitigate this effect and warrant empirical investigation.

Finally, the computational overhead of LLM inference introduces a practical constraint absent in classical memetic algorithms. Although the framework mitigates this through library reuse with LLM calls ceasing once ${|L|=L}_{max}$∣, the latency of individual inference calls may be prohibitive in time-sensitive or resource-constrained applications. Lightweight surrogate models trained on accumulated prompt-response pairs, or retrieval-augmented generation over the existing library, could reduce this overhead while preserving operator diversity. These considerations highlight that the transition to fourth-generation memetic algorithms involves not only algorithmic novelty but also practical engineering challenges that require dedicated investigation.

## 8. Conclusions

This paper presented a fourth-generation memetic framework in which large language models are embedded directly into the optimization loop as adaptive generators of local search operators. The framework addresses a fundamental limitation of existing memetic algorithms: the reliance on manually designed, problem-specific meme pools fixed prior to execution. It enables on-demand meme synthesis conditioned on a structured representation of the current search state. The proposed state vector encodes eight complementary metrics capturing convergence dynamics, population geometry, fitness heterogeneity, and landscape multimodality, providing the LLM with sufficient context to generate operators adapted to the evolving search regime. A two-stage novelty filtering mechanism combining semantic and implementation-level similarity assessment ensures that the dynamically growing meme library accumulates genuinely diverse operators, while a cooldown-regulated triggering policy coordinates periodic and stagnation-based generation without redundant LLM calls.

Computational experiments on the CEC 2017 benchmark suite demonstrate that the proposed LLM-based variants AIM-PSO and AIM-WOAs consistently outperform their non-LLM counterparts across all baseline categories, including pure global search, single-meme and randomly selecting multi-memetic variants. AIM-WOA achieved the strongest overall performance with 15 pairwise wins (51.7%), followed by AIM-PSO with 8 wins (27.6%), confirming that the advantage of the framework is robust to the choice of the underlying global optimizer. Meme similarity analysis further validates the generation mechanism: LLM-generated operators are structurally distinct from classical local search baselines such as Nelder–Mead, exhibit meaningful variation across different search regimes, and display stable behavior when conditioned on similar state vectors. This collectively supports the hypothesis that LLMs are capable of generating genuinely novel and contextually appropriate optimization strategies.

The present work constitutes a concrete algorithmic realization of the fourth-generation memetic paradigm, which has previously remained largely conceptual. By formalizing the co-evolutionary relationship between candidate solutions and meme operators, and by grounding meme generation in empirical optimization feedback rather than offline design, the framework advances memetic computation toward a fully adaptive and general-purpose optimization methodology.

Several directions for future work follow naturally from the present framework. The fixed trigger interval Δ could be replaced by an adaptive policy that initiates LLM calls in response to detected regime changes in $E_{t}$, while meme mutation, in other words prompting the LLM to refine an existing library operator based on its accumulated performance statistics, would introduce a Lamarckian element into library evolution. A hierarchical library organization indexed by search regime at the time of meme generation would enable more targeted operator retrieval. Finally, systematic evaluation across LLM architectures and model scales would allow the influence of model capability on meme quality, operator diversity, and code validity to be characterized, bridging the optimization and artificial intelligence perspectives on the proposed framework.

## Figures and Tables

**Table 1:** Experimental results for the considered optimization algorithms.

Benchmark Function	PSO	WOA	PSO-NM	WOA-NM	MMPSO	MMWOA	AIM-PSO	AIM-WOA
$F_{1}$	$\bar{F}=8.1\times 10^{9}$	$\bar{F}=9.4\times 10^{6}$	$\bar{F}=1.1\times 10^{4}$	$\bar{F}=1.5\times 10^{7}$	$\bar{F}=1.1\times 10^{4}$	$\bar{F}=1.7\times 10^{6}$	$\bar{F}=6.9\times 10^{3}$	$\bar{F}=1.8\times 10^{3}$
	$\sigma =5.5\times 10^{9}$	$\sigma =4.1\times 10^{6}$	$\sigma =7.9\times 10^{3}$	$\sigma =2.2\times 10^{7}$	$\sigma =7.4\times 10^{3}$	$\sigma =8.7\times 10^{5}$	$\sigma =7.8\times 10^{3}$	$\sigma =3.1\times 10^{3}$
	$F_{min}=1.8\times 10^{4}$	$F_{min}=4.8\times 10^{6}$	$F_{min}=5.6\times 10^{2}$	$F_{min}=2.0\times 10^{6}$	$F_{min}=1.7\times 10^{2}$	$F_{min}=6.7\times 10^{5}$	$F_{min}=1.0\times 10^{2}$	$F_{min}=1.0\times 10^{2}$
$F_{2}$	$\bar{F}=3.1\times 10^{3}$	$\bar{F}=5.4\times 10^{2}$	$\bar{F}=2.0\times 10^{2}$	$\bar{F}=1.2\times 10^{3}$	$\bar{F}=2.0\times 10^{2}$	$\bar{F}=2.1\times 10^{2}$	$\bar{F}=2.0\times 10^{2}$	$\bar{F}=2.0\times 10^{2}$
	$\sigma =2.8\times 10^{3}$	$\sigma =3.8\times 10^{2}$	$\sigma =2.7\times 10^{-14}$	$\sigma =1.1\times 10^{3}$	$\sigma =1.6\times 10^{-14}$	$\sigma =2.9\times 10^{0}$	$\sigma =2.1\times 10^{-14}$	$\sigma =6.1\times 10^{-8}$
	$F_{min}=2.0\times 10^{2}$	$F_{min}=3.1\times 10^{2}$	$F_{min}=2.0\times 10^{2}$	$F_{min}=3.1\times 10^{2}$	$F_{min}=2.0\times 10^{2}$	$F_{min}=2.0\times 10^{2}$	$F_{min}=2.0\times 10^{2}$	$F_{min}=2.0\times 10^{2}$
$F_{3}$	$\bar{F}=8.8\times 10^{2}$	$\bar{F}=4.4\times 10^{2}$	$\bar{F}=3.4\times 10^{2}$	$\bar{F}=4.6\times 10^{2}$	$\bar{F}=3.5\times 10^{2}$	$\bar{F}=3.8\times 10^{2}$	$\bar{F}=3.3\times 10^{2}$	$\bar{F}=3.1\times 10^{2}$
	$\sigma =5.1\times 10^{2}$	$\sigma =8.3\times 10^{1}$	$\sigma =1.9\times 10^{1}$	$\sigma =5.6\times 10^{1}$	$\sigma =2.8\times 10^{1}$	$\sigma =3.9\times 10^{1}$	$\sigma =9.3\times 10^{0}$	$\sigma =1.5\times 10^{1}$
	$F_{min}=3.3\times 10^{2}$	$F_{min}=3.4\times 10^{2}$	$F_{min}=3.3\times 10^{2}$	$F_{min}=3.3\times 10^{2}$	$F_{min}=3.3\times 10^{2}$	$F_{min}=3.3\times 10^{2}$	$F_{min}=3.0\times 10^{2}$	$F_{min}=3.0\times 10^{2}$
$F_{4}$	$\bar{F}=1.1\times 10^{4}$	$\bar{F}=1.6\times 10^{3}$	$\bar{F}=5.6\times 10^{2}$	$\bar{F}=1.5\times 10^{3}$	$\bar{F}=5.5\times 10^{2}$	$\bar{F}=8.6\times 10^{2}$	$\bar{F}=4.8\times 10^{2}$	$\bar{F}=4.3\times 10^{2}$
	$\sigma =5.5\times 10^{3}$	$\sigma =3.4\times 10^{2}$	$\sigma =6.4\times 10^{1}$	$\sigma =3.1\times 10^{2}$	$\sigma =4.1\times 10^{1}$	$\sigma =1.4\times 10^{2}$	$\sigma =2.1\times 10^{1}$	$\sigma =6.5\times 10^{0}$
	$F_{min}=2.6\times 10^{3}$	$F_{min}=1.1\times 10^{3}$	$F_{min}=4.7\times 10^{2}$	$F_{min}=1.1\times 10^{3}$	$F_{min}=4.9\times 10^{2}$	$F_{min}=6.7\times 10^{2}$	$F_{min}=4.4\times 10^{2}$	$F_{min}=4.2\times 10^{2}$
$F_{5}$	$\bar{F}=5.0\times 10^{2}$	$\bar{F}=5.0\times 10^{2}$	$\bar{F}=5.0\times 10^{2}$	$\bar{F}=5.0\times 10^{2}$	$\bar{F}=5.0\times 10^{2}$	$\bar{F}=5.0\times 10^{2}$	$\bar{F}=5.0\times 10^{2}$	$\bar{F}=5.0\times 10^{2}$
	$\sigma =3.1\times 10^{-4}$	$\sigma =6.0\times 10^{-3}$	$\sigma =6.4\times 10^{-4}$	$\sigma =1.1\times 10^{-3}$	$\sigma =7.4\times 10^{-4}$	$\sigma =3.4\times 10^{-3}$	$\sigma =1.9\times 10^{-4}$	$\sigma =7.5\times 10^{-4}$
	$F_{min}=5.0\times 10^{2}$	$F_{min}=5.0\times 10^{2}$	$F_{min}=5.0\times 10^{2}$	$F_{min}=5.0\times 10^{2}$	$F_{min}=5.0\times 10^{2}$	$F_{min}=5.0\times 10^{2}$	$F_{min}=5.0\times 10^{2}$	$F_{min}=5.0\times 10^{2}$
$F_{6}$	$\bar{F}=3.7\times 10^{5}$	$\bar{F}=3.1\times 10^{3}$	$\bar{F}=7.5\times 10^{2}$	$\bar{F}=2.3\times 10^{3}$	$\bar{F}=7.6\times 10^{2}$	$\bar{F}=1.1\times 10^{3}$	$\bar{F}=7.1\times 10^{2}$	$\bar{F}=6.7\times 10^{2}$
	$\sigma =2.1\times 10^{5}$	$\sigma =7.1\times 10^{2}$	$\sigma =4.2\times 10^{1}$	$\sigma =1.6\times 10^{3}$	$\sigma =4.0\times 10^{1}$	$\sigma =1.8\times 10^{2}$	$\sigma =2.4\times 10^{1}$	$\sigma =1.4\times 10^{1}$
	$F_{min}=4.8\times 10^{4}$	$F_{min}=2.1\times 10^{3}$	$F_{min}=6.8\times 10^{2}$	$F_{min}=9.5\times 10^{2}$	$F_{min}=7.0\times 10^{2}$	$F_{min}=9.3\times 10^{2}$	$F_{min}=6.7\times 10^{2}$	$F_{min}=6.3\times 10^{2}$
$F_{7}$	$\bar{F}=9.5\times 10^{2}$	$\bar{F}=1.3\times 10^{3}$	$\bar{F}=9.7\times 10^{2}$	$\bar{F}=1.2\times 10^{3}$	$\bar{F}=9.7\times 10^{2}$	$\bar{F}=1.2\times 10^{3}$	$\bar{F}=9.9\times 10^{2}$	$\bar{F}=1.2\times 10^{3}$
	$\sigma =7.7\times 10^{1}$	$\sigma =1.1\times 10^{2}$	$\sigma =6.5\times 10^{1}$	$\sigma =1.5\times 10^{2}$	$\sigma =8.3\times 10^{1}$	$\sigma =1.2\times 10^{2}$	$\sigma =5.1\times 10^{1}$	$\sigma =1.2\times 10^{2}$
	$F_{min}=8.7\times 10^{2}$	$F_{min}=1.1\times 10^{3}$	$F_{min}=8.7\times 10^{2}$	$F_{min}=9.4\times 10^{2}$	$F_{min}=8.5\times 10^{2}$	$F_{min}=1.0\times 10^{3}$	$F_{min}=8.8\times 10^{2}$	$F_{min}=9.5\times 10^{2}$
$F_{8}$	$\bar{F}=8.1\times 10^{2}$	$\bar{F}=8.1\times 10^{2}$	$\bar{F}=8.1\times 10^{2}$	$\bar{F}=8.2\times 10^{2}$	$\bar{F}=8.1\times 10^{2}$	$\bar{F}=8.2\times 10^{2}$	$\bar{F}=8.1\times 10^{2}$	$\bar{F}=8.2\times 10^{2}$
	$\sigma =3.8\times 10^{0}$	$\sigma =7.1\times 10^{0}$	$\sigma =5.1\times 10^{0}$	$\sigma =1.1\times 10^{1}$	$\sigma =5.9\times 10^{0}$	$\sigma =6.9\times 10^{0}$	$\sigma =6.2\times 10^{0}$	$\sigma =7.4\times 10^{0}$
	$F_{min}=8.1\times 10^{2}$	$F_{min}=8.1\times 10^{2}$	$F_{min}=8.1\times 10^{2}$	$F_{min}=8.0\times 10^{2}$	$F_{min}=8.0\times 10^{2}$	$F_{min}=8.1\times 10^{2}$	$F_{min}=8.0\times 10^{2}$	$F_{min}=8.1\times 10^{2}$
$F_{9}$	$\bar{F}=4.3\times 10^{3}$	$\bar{F}=5.8\times 10^{3}$	$\bar{F}=4.5\times 10^{3}$	$\bar{F}=5.6\times 10^{3}$	$\bar{F}=4.3\times 10^{3}$	$\bar{F}=5.6\times 10^{3}$	$\bar{F}=3.7\times 10^{3}$	$\bar{F}=4.5\times 10^{3}$
	$\sigma =6.2\times 10^{2}$	$\sigma =6.8\times 10^{2}$	$\sigma =8.3\times 10^{2}$	$\sigma =9.2\times 10^{2}$	$\sigma =4.3\times 10^{2}$	$\sigma =6.9\times 10^{2}$	$\sigma =3.8\times 10^{2}$	$\sigma =7.7\times 10^{2}$
	$F_{min}=2.9\times 10^{3}$	$F_{min}=4.2\times 10^{3}$	$F_{min}=3.2\times 10^{3}$	$F_{min}=4.2\times 10^{3}$	$F_{min}=3.5\times 10^{3}$	$F_{min}=4.8\times 10^{3}$	$F_{min}=2.9\times 10^{3}$	$F_{min}=3.6\times 10^{3}$
$F_{10}$	$\bar{F}=5.9\times 10^{5}$	$\bar{F}=1.3\times 10^{5}$	$\bar{F}=1.7\times 10^{4}$	$\bar{F}=8.6\times 10^{4}$	$\bar{F}=8.5\times 10^{3}$	$\bar{F}=9.5\times 10^{4}$	$\bar{F}=6.4\times 10^{3}$	$\bar{F}=6.7\times 10^{3}$
	$\sigma =1.1\times 10^{6}$	$\sigma =3.7\times 10^{4}$	$\sigma =2.7\times 10^{4}$	$\sigma =2.7\times 10^{4}$	$\sigma =1.3\times 10^{4}$	$\sigma =2.2\times 10^{4}$	$\sigma =6.6\times 10^{3}$	$\sigma =6.1\times 10^{3}$
	$F_{min}=9.1\times 10^{4}$	$F_{min}=4.6\times 10^{4}$	$F_{min}=1.3\times 10^{3}$	$F_{min}=5.7\times 10^{4}$	$F_{min}=1.2\times 10^{3}$	$F_{min}=5.7\times 10^{4}$	$F_{min}=1.1\times 10^{3}$	$F_{min}=1.1\times 10^{3}$
$F_{11}$	$\bar{F}=5.4\times 10^{8}$	$\bar{F}=3.7\times 10^{7}$	$\bar{F}=2.8\times 10^{6}$	$\bar{F}=9.7\times 10^{6}$	$\bar{F}=2.2\times 10^{6}$	$\bar{F}=1.3\times 10^{7}$	$\bar{F}=1.1\times 10^{4}$	$\bar{F}=1.2\times 10^{4}$
	$\sigma =8.4\times 10^{8}$	$\sigma =2.6\times 10^{7}$	$\sigma =2.4\times 10^{6}$	$\sigma =1.1\times 10^{7}$	$\sigma =3.2\times 10^{6}$	$\sigma =1.1\times 10^{7}$	$\sigma =3.6\times 10^{3}$	$\sigma =5.4\times 10^{3}$
	$F_{min}=1.2\times 10^{5}$	$F_{min}=5.5\times 10^{6}$	$F_{min}=6.7\times 10^{4}$	$F_{min}=7.5\times 10^{5}$	$F_{min}=2.2\times 10^{4}$	$F_{min}=2.7\times 10^{6}$	$F_{min}=3.8\times 10^{3}$	$F_{min}=4.4\times 10^{3}$
$F_{12}$	$\bar{F}=2.2\times 10^{8}$	$\bar{F}=3.6\times 10^{6}$	$\bar{F}=8.6\times 10^{4}$	$\bar{F}=7.7\times 10^{5}$	$\bar{F}=7.0\times 10^{4}$	$\bar{F}=6.8\times 10^{5}$	$\bar{F}=1.2\times 10^{4}$	$\bar{F}=1.6\times 10^{3}$
	$\sigma =4.5\times 10^{8}$	$\sigma =1.4\times 10^{6}$	$\sigma =2.6\times 10^{4}$	$\sigma =1.3\times 10^{6}$	$\sigma =2.5\times 10^{4}$	$\sigma =9.8\times 10^{5}$	$\sigma =6.0\times 10^{3}$	$\sigma =6.7\times 10^{2}$
	$F_{min}=1.1\times 10^{5}$	$F_{min}=1.6\times 10^{6}$	$F_{min}=4.8\times 10^{4}$	$F_{min}=6.0\times 10^{4}$	$F_{min}=2.6\times 10^{4}$	$F_{min}=7.7\times 10^{4}$	$F_{min}=1.5\times 10^{3}$	$F_{min}=1.2\times 10^{3}$
$F_{13}$	$\bar{F}=1.1\times 10^{5}$	$\bar{F}=1.4\times 10^{6}$	$\bar{F}=1.9\times 10^{5}$	$\bar{F}=2.2\times 10^{5}$	$\bar{F}=1.4\times 10^{5}$	$\bar{F}=6.6\times 10^{5}$	$\bar{F}=6.7\times 10^{4}$	$\bar{F}=2.9\times 10^{5}$
	$\sigma =7.2\times 10^{4}$	$\sigma =1.3\times 10^{6}$	$\sigma =1.1\times 10^{5}$	$\sigma =3.4\times 10^{6}$	$\sigma =9.7\times 10^{4}$	$\sigma =6.5\times 10^{5}$	$\sigma =6.1\times 10^{4}$	$\sigma =2.9\times 10^{5}$
	$F_{min}=3.6\times 10^{4}$	$F_{min}=2.7\times 10^{5}$	$F_{min}=4.8\times 10^{4}$	$F_{min}=2.4\times 10^{5}$	$F_{min}=1.4\times 10^{4}$	$F_{min}=1.8\times 10^{5}$	$F_{min}=2.3\times 10^{3}$	$F_{min}=2.5\times 10^{3}$
$F_{14}$	$\bar{F}=2.9\times 10^{7}$	$\bar{F}=8.1\times 10^{5}$	$\bar{F}=2.3\times 10^{7}$	$\bar{F}=1.2\times 10^{5}$	$\bar{F}=1.1\times 10^{5}$	$\bar{F}=1.7\times 10^{5}$	$\bar{F}=1.3\times 10^{4}$	$\bar{F}=1.6\times 10^{4}$
	$\sigma =7.3\times 10^{7}$	$\sigma =5.1\times 10^{5}$	$\sigma =6.8\times 10^{7}$	$\sigma =4.9\times 10^{4}$	$\sigma =5.7\times 10^{4}$	$\sigma =1.4\times 10^{5}$	$\sigma =8.3\times 10^{3}$	$\sigma =9.7\times 10^{3}$
	$F_{min}=9.5\times 10^{4}$	$F_{min}=1.9\times 10^{5}$	$F_{min}=3.3\times 10^{4}$	$F_{min}=5.5\times 10^{4}$	$F_{min}=2.4\times 10^{4}$	$F_{min}=6.9\times 10^{4}$	$F_{min}=2.8\times 10^{3}$	$F_{min}=2.3\times 10^{3}$
$F_{15}$	$\bar{F}=9.1\times 10^{6}$	$\bar{F}=4.8\times 10^{5}$	$\bar{F}=3.5\times 10^{3}$	$\bar{F}=6.9\times 10^{5}$	$\bar{F}=2.9\times 10^{3}$	$\bar{F}=2.4\times 10^{4}$	$\bar{F}=2.1\times 10^{3}$	$\bar{F}=1.9\times 10^{3}$
	$\sigma =2.8\times 10^{7}$	$\sigma =6.1\times 10^{5}$	$\sigma =1.2\times 10^{3}$	$\sigma =6.3\times 10^{5}$	$\sigma =1.7\times 10^{3}$	$\sigma =1.2\times 10^{4}$	$\sigma =4.2\times 10^{2}$	$\sigma =3.9\times 10^{2}$
	$F_{min}=6.1\times 10^{3}$	$F_{min}=2.3\times 10^{4}$	$F_{min}=1.9\times 10^{3}$	$F_{min}=4.4\times 10^{3}$	$F_{min}=1.5\times 10^{3}$	$F_{min}=4.6\times 10^{3}$	$F_{min}=1.5\times 10^{3}$	$F_{min}=1.5\times 10^{3}$
$F_{16}$	$\bar{F}=2.5\times 10^{5}$	$\bar{F}=5.9\times 10^{4}$	$\bar{F}=2.5\times 10^{3}$	$\bar{F}=2.9\times 10^{4}$	$\bar{F}=2.3\times 10^{3}$	$\bar{F}=1.6\times 10^{4}$	$\bar{F}=2.1\times 10^{3}$	$\bar{F}=1.8\times 10^{3}$
	$\sigma =6.9\times 10^{5}$	$\sigma =3.5\times 10^{4}$	$\sigma =4.6\times 10^{2}$	$\sigma =1.2\times 10^{4}$	$\sigma =5.3\times 10^{2}$	$\sigma =8.7\times 10^{3}$	$\sigma =4.2\times 10^{3}$	$\sigma =3.7\times 10^{3}$
	$F_{min}=1.2\times 10^{4}$	$F_{min}=9.3\times 10^{3}$	$F_{min}=1.6\times 10^{3}$	$F_{min}=9.2\times 10^{3}$	$F_{min}=1.6\times 10^{3}$	$F_{min}=4.3\times 10^{3}$	$F_{min}=1.6\times 10^{3}$	$F_{min}=1.6\times 10^{3}$
$F_{17}$	$\bar{F}=9.8\times 10^{4}$	$\bar{F}=7.1\times 10^{5}$	$\bar{F}=8.3\times 10^{4}$	$\bar{F}=3.8\times 10^{5}$	$\bar{F}=8.9\times 10^{4}$	$\bar{F}=3.1\times 10^{5}$	$\bar{F}=3.7\times 10^{4}$	$\bar{F}=1.6\times 10^{5}$
	$\sigma =7.6\times 10^{4}$	$\sigma =6.4\times 10^{5}$	$\sigma =6.2\times 10^{4}$	$\sigma =2.5\times 10^{5}$	$\sigma =6.5\times 10^{4}$	$\sigma =2.1\times 10^{5}$	$\sigma =2.0\times 10^{4}$	$\sigma =1.1\times 10^{5}$
	$F_{min}=1.2\times 10^{4}$	$F_{min}=1.1\times 10^{5}$	$F_{min}=1.7{\times 10}^{4}$	$F_{min}=1.0\times 10^{5}$	$F_{min}=1.7\times 10^{4}$	$F_{min}=5.2\times 10^{4}$	$F_{min}=9.9\times 10^{3}$	$F_{min}=2.9\times 10^{4}$
$F_{18}$	$\bar{F}=2.9\times 10^{9}$	$\bar{F}=1.3\times 10^{5}$	$\bar{F}=1.1\times 10^{7}$	$\bar{F}=7.4\times 10^{4}$	$\bar{F}=8.4\times 10^{4}$	$\bar{F}=7.6\times 10^{4}$	$\bar{F}=3.4\times 10^{4}$	$\bar{F}=8.8\times 10^{3}$
	$\sigma =5.9\times 10^{9}$	$\sigma =6.3\times 10^{4}$	$\sigma =3.5\times 10^{7}$	$\sigma =3.2\times 10^{4}$	$\sigma =4.9\times 10^{4}$	$\sigma =4.1\times 10^{4}$	$\sigma =2.7\times 10^{4}$	$\sigma =9.8\times 10^{3}$
	$F_{min}=3.4\times 10^{4}$	$F_{min}=3.4\times 10^{4}$	$F_{min}=1.6\times 10^{4}$	$F_{min}=2.8\times 10^{4}$	$F_{min}=1.4\times 10^{4}$	$F_{min}=2.1\times 10^{4}$	$F_{min}=2.4\times 10^{3}$	$F_{min}=1.8\times 10^{3}$
$F_{19}$	$\bar{F}=2.4\times 10^{3}$	$\bar{F}=4.1\times 10^{3}$	$\bar{F}=2.4\times 10^{3}$	$\bar{F}=3.8\times 10^{3}$	$\bar{F}=2.4\times 10^{3}$	$\bar{F}=3.8\times 10^{3}$	$\bar{F}=2.4\times 10^{3}$	$\bar{F}=2.1\times 10^{3}$
	$\sigma =3.9\times 10^{2}$	$\sigma =9.3\times 10^{2}$	$\sigma =3.4\times 10^{2}$	$\sigma =5.5\times 10^{2}$	$\sigma =3.4\times 10^{2}$	$\sigma =7.2\times 10^{2}$	$\sigma =3.0\times 10^{2}$	$\sigma =2.4\times 10^{2}$
	$F_{min}=1.9\times 10^{3}$	$F_{min}=3.1\times 10^{3}$	$F_{min}=1.9\times 10^{3}$	$F_{min}=3.0\times 10^{3}$	$F_{min}=1.9\times 10^{3}$	$F_{min}=2.6\times 10^{3}$	$F_{min}=1.9\times 10^{3}$	$F_{min}=1.9\times 10^{3}$
$F_{20}$	$\bar{F}=6.9\times 10^{3}$	$\bar{F}=2.9\times 10^{3}$	$\bar{F}=2.3\times 10^{3}$	$\bar{F}=2.9\times 10^{3}$	$\bar{F}=2.3\times 10^{3}$	$\bar{F}=2.5\times 10^{3}$	$\bar{F}=2.2\times 10^{3}$	$\bar{F}=2.2\times 10^{3}$
	$\sigma =3.5\times 10^{3}$	$\sigma =7.7\times 10^{2}$	$\sigma =1.2\times 10^{2}$	$\sigma =6.0\times 10^{2}$	$\sigma =1.3\times 10^{2}$	$\sigma =4.2\times 10^{2}$	$\sigma =1.0\times 10^{2}$	$\sigma =7.9\times 10^{1}$
	$F_{min}=3.4\times 10^{3}$	$F_{min}=2.1\times 10^{3}$	$F_{min}=2.1\times 10^{3}$	$F_{min}=2.2\times 10^{3}$	$F_{min}=2.1\times 10^{3}$	$F_{min}=2.1\times 10^{3}$	$F_{min}=2.1\times 10^{3}$	$F_{min}=2.1\times 10^{3}$
$F_{21}$	$\bar{F}=2.5\times 10^{3}$	$\bar{F}=2.6\times 10^{3}$	$\bar{F}=2.4\times 10^{3}$	$\bar{F}=2.8\times 10^{3}$	$\bar{F}=2.4\times 10^{3}$	$\bar{F}=2.7\times 10^{3}$	$\bar{F}=2.4\times 10^{3}$	$\bar{F}=2.7\times 10^{3}$
	$\sigma =9.1\times 10^{1}$	$\sigma =1.9\times 10^{2}$	$\sigma =6.1\times 10^{1}$	$\sigma =5.1\times 10^{2}$	$\sigma =4.4\times 10^{1}$	$\sigma =2.5\times 10^{2}$	$\sigma =3.1\times 10^{1}$	$\sigma =2.7\times 10^{12}$
	$F_{min}=2.4\times 10^{3}$	$F_{min}=2.4\times 10^{3}$	$F_{min}=2.3\times 10^{3}$	$F_{min}=2.4\times 10^{3}$	$F_{min}=2.3\times 10^{3}$	$F_{min}=2.4\times 10^{3}$	$F_{min}=2.3\times 10^{3}$	$F_{min}=2.4\times 10^{3}$
$F_{22}$	$\bar{F}=1.7\times 10^{4}$	$\bar{F}=2.9\times 10^{3}$	$\bar{F}=2.4\times 10^{3}$	$\bar{F}=2.8\times 10^{3}$	$\bar{F}=2.5\times 10^{3}$	$\bar{F}=2.6\times 10^{3}$	$\bar{F}=2.5\times 10^{3}$	$\bar{F}=2.5\times 10^{3}$
	$\sigma =4.1\times 10^{3}$	$\sigma =4.9\times 10^{2}$	$\sigma =7.8\times 10^{1}$	$\sigma =7.0\times 10^{2}$	$\sigma =2.2\times 10^{2}$	$\sigma =2.1\times 10^{2}$	$\sigma =1.1\times 10^{2}$	$\sigma =7.1\times 10^{1}$
	$F_{min}=7.8\times 10^{3}$	$F_{min}=2.6\times 10^{3}$	$F_{min}=2.3\times 10^{3}$	$F_{min}=2.5\times 10^{3}$	$F_{min}=2.3\times 10^{3}$	$F_{min}=2.5\times 10^{3}$	$F_{min}=2.4\times 10^{3}$	$F_{min}=2.4\times 10^{3}$
$F_{23}$	$\bar{F}=1.2\times 10^{4}$	$\bar{F}=2.7\times 10^{3}$	$\bar{F}=2.5\times 10^{3}$	$\bar{F}=2.8\times 10^{3}$	$\bar{F}=2.5\times 10^{3}$	$\bar{F}=2.6\times 10^{3}$	$\bar{F}=2.5\times 10^{3}$	$\bar{F}=2.5\times 10^{3}$
	$\sigma =2.5\times 10^{3}$	$\sigma =2.6\times 10^{2}$	$\sigma =8.6\times 10^{1}$	$\sigma =3.9\times 10^{2}$	$\sigma =1.1\times 10^{2}$	$\sigma =1.3\times 10^{2}$	$\sigma =4.2\times 10^{1}$	$\sigma =3.6\times 10^{1}$
	$F_{min}=6.8\times 10^{3}$	$F_{min}=2.6\times 10^{3}$	$F_{min}=2.5\times 10^{3}$	$F_{min}=2.6\times 10^{3}$	$F_{min}=2.5\times 10^{3}$	$F_{min}=2.6\times 10^{3}$	$F_{min}=2.5\times 10^{3}$	$F_{min}=2.5\times 10^{3}$
$F_{24}$	$\bar{F}=3.6\times 10^{3}$	$\bar{F}=2.9\times 10^{3}$	$\bar{F}=2.8\times 10^{3}$	$\bar{F}=2.9\times 10^{3}$	$\bar{F}=2.8\times 10^{3}$	$\bar{F}=2.9\times 10^{3}$	$\bar{F}=2.8\times 10^{3}$	$\bar{F}=2.8\times 10^{3}$
	$\sigma =2.1\times 10^{2}$	$\sigma =5.9\times 10^{1}$	$\sigma =3.0\times 10^{1}$	$\sigma =4.0\times 10^{1}$	$\sigma =5.3\times 10^{0}$	$\sigma =4.6\times 10^{1}$	$\sigma =1.9\times 10^{1}$	$\sigma =1.7\times 10^{1}$
	$F_{min}=2.8\times 10^{3}$	$F_{min}=2.8\times 10^{3}$	$F_{min}=2.8\times 10^{3}$	$F_{min}=3.1\times 10^{3}$	$F_{min}=2.8\times 10^{3}$	$F_{min}=2.8\times 10^{3}$	$F_{min}=2.8\times 10^{3}$	$F_{min}=2.8\times 10^{3}$
$F_{25}$	$\bar{F}=3.6\times 10^{3}$	$\bar{F}=3.3\times 10^{3}$	$\bar{F}=3.6\times 10^{3}$	$\bar{F}=3.4\times 10^{3}$	$\bar{F}=3.4\times 10^{3}$	$\bar{F}=3.4\times 10^{3}$	$\bar{F}=3.3\times 10^{3}$	$\bar{F}=3.3\times 10^{3}$
	$\sigma =1.2\times 10^{2}$	$\sigma =9.9\times 10^{0}$	$\sigma =3.3\times 10^{2}$	$\sigma =1.6\times 10^{1}$	$\sigma =8.4\times 10^{1}$	$\sigma =2.9\times 10^{1}$	$\sigma =3.1\times 10^{-2}$	$\sigma =1.5\times 10^{1}$
	$F_{min}=3.4\times 10^{3}$	$F_{min}=3.3\times 10^{3}$	$F_{min}=3.3\times 10^{3}$	$F_{min}=3.3\times 10^{3}$	$F_{min}=3.3\times 10^{3}$	$F_{min}=3.3\times 10^{3}$	$F_{min}=3.3\times 10^{3}$	$F_{min}=3.2\times 10^{3}$
$F_{26}$	$\bar{F}=3.2\times 10^{3}$	$\bar{F}=3.3\times 10^{3}$	$\bar{F}=3.2\times 10^{3}$	$\bar{F}=3.3\times 10^{3}$	$\bar{F}=3.2\times 10^{3}$	$\bar{F}=3.3\times 10^{3}$	$\bar{F}=3.1\times 10^{3}$	$\bar{F}=3.2\times 10^{3}$
	$\sigma =5.9\times 10^{1}$	$\sigma =1.2\times 10^{2}$	$\sigma =4.4\times 10^{1}$	$\sigma =8.3\times 10^{1}$	$\sigma =3.1\times 10^{1}$	$\sigma =1.5\times 10^{2}$	$\sigma =2.9\times 10^{1}$	$\sigma =3.9\times 10^{1}$
	$F_{min}=3.1\times 10^{3}$	$F_{min}=3.1\times 10^{3}$	$F_{min}=3.1\times 10^{3}$	$F_{min}=3.1\times 10^{3}$	$F_{min}=3.1\times 10^{3}$	$F_{min}=3.1\times 10^{3}$	$F_{min}=3.1\times 10^{3}$	$F_{min}=3.1\times 10^{3}$
$F_{27}$	$\bar{F}=3.7\times 10^{3}$	$\bar{F}=3.1\times 10^{3}$	$\bar{F}=2.7\times 10^{3}$	$\bar{F}=3.2\times 10^{3}$	$\bar{F}=2.7\times 10^{3}$	$\bar{F}=3.1\times 10^{3}$	$\bar{F}=2.7\times 10^{3}$	$\bar{F}=2.7\times 10^{3}$
	$\sigma =4.1\times 10^{2}$	$\sigma =7.3\times 10^{1}$	$\sigma =1.1\times 10^{1}$	$\sigma =4.0\times 10^{1}$	$\sigma =1.1\times 10^{1}$	$\sigma =1.9\times 10^{2}$	$\sigma =1.2\times 10^{1}$	$\sigma =3.9\times 10^{-4}$
	$F_{min}=2.9\times 10^{3}$	$F_{min}=2.9\times 10^{3}$	$F_{min}=2.7\times 10^{3}$	$F_{min}=3.1\times 10^{3}$	$F_{min}=2.7\times 10^{3}$	$F_{min}=2.7\times 10^{3}$	$F_{min}=2.7\times 10^{3}$	$F_{min}=2.7\times 10^{3}$
$F_{28}$	$\bar{F}=2.2\times 10^{8}$	$\bar{F}=7.2\times 10^{5}$	$\bar{F}=3.4\times 10^{6}$	$\bar{F}=7.6\times 10^{6}$	$\bar{F}=1.1\times 10^{4}$	$\bar{F}=7.4\times 10^{4}$	$\bar{F}=7.9\times 10^{3}$	$\bar{F}=6.6\times 10^{3}$
	$\sigma =3.1\times 10^{8}$	$\sigma =2.1\times 10^{6}$	$\sigma =1.5\times 10^{8}$	$\sigma =2.9\times 10^{7}$	$\sigma =1.2\times 10^{4}$	$\sigma =5.5\times 10^{4}$	$\sigma =2.0\times 10^{3}$	$\sigma =1.6\times 10^{3}$
	$F_{min}=3.7\times 10^{4}$	$F_{min}=4.3\times 10^{4}$	$F_{min}=4.9\times 10^{3}$	$F_{min}=5.0\times 10^{4}$	$F_{min}=5.6\times 10^{3}$	$F_{min}=2.5\times 10^{4}$	$F_{min}=4.4\times 10^{3}$	$F_{min}=4.7\times 10^{3}$
$F_{29}$	$\bar{F}=1.2\times 10^{9}$	$\bar{F}=9.3\times 10^{6}$	$\bar{F}=1.5\times 10^{8}$	$\bar{F}=1.7\times 10^{7}$	$\bar{F}=2.9\times 10^{5}$	$\bar{F}=4.9\times 10^{6}$	$\bar{F}=4.4\times 10^{4}$	$\bar{F}=2.3\times 10^{4}$
	$\sigma =2.6\times 10^{9}$	$\sigma =1.4\times 10^{7}$	$\sigma =3.3\times 10^{8}$	$\sigma =2.8\times 10^{7}$	$\sigma =7.6\times 10^{5}$	$\sigma =5.4\times 10^{6}$	$\sigma =2.3\times 10^{4}$	$\sigma =2.1\times 10^{4}$
	$F_{min}=8.9\times 10^{4}$	$F_{min}=3.7\times 10^{5}$	$F_{min}=4.8\times 10^{4}$	$F_{min}=1.5\times 10^{5}$	$F_{min}=2.4\times 10^{4}$	$F_{min}=1.4\times 10^{5}$	$F_{min}=6.3\times 10^{3}$	$F_{min}=5.2\times 10^{3}$
Rank	6.41	6.51	3.96	6.31	3.24	5.34	1.86	2.34

Bold denotes the best result per function.

**Table 2:** Composite efficiency scores of LLM-generated meme operators averaged across five benchmark functions; lower values indicate better performance.

Top 5 Generated Memes	Composite Score	Last 5 Generated Memes	Composite Score
$m_{1}^{13}$	0.0445	$m_{3}^{11}$	0.7685
$m_{1}^{1}$	0.0761	$m_{1}^{17}$	0.7723
$m_{3}^{15}$	0.1032	$m_{3}^{17}$	0.7789
$m_{1}^{9}$	0.1106	$m_{3}^{9}$	0.8108
$m_{1}^{29}$	0.1165	$m_{3}^{25}$	0.8413

## Data Availability

All experimental results are available from the corresponding author upon request.
